# Concurrent Central and Autonomic Nervous System Involvement in Varicella-Zoster Virus Infection in an Immunocompetent Patient: A Case-Based Mechanistic Analysis

**DOI:** 10.3390/idr18030058

**Published:** 2026-06-15

**Authors:** Jordan Pyatt, Carlos A. Umaña Mejía, Justice Cruz, Fernando Baires, Helen Hoffman, Joanne Cordero Guerra, Miguel Sierra-Hoffman, Heike Hesse, Amy C. Madril

**Affiliations:** 1 College of Osteopathic Medicine, Sam Houston State University, Conroe, TX 77304, USA; jep079@shsu.com; 2 Facultad de Medicina, Universidad Autónoma de Guadalajara, Guadalajara 45129, Mexico; umanacarlos72@gmail.com; 3 Victoria College, Victoria, TX 77901, USA; leonardj@tamuv.edu; 4 Facultad de Ciencias Médicas, Universidad Nacional Autónoma de Honduras, Tegucigalpa 11101, Honduras; ferbaires93@hotmail.com; 5 Facultad de Ciencias de la Salud, Universidad Latina de Costa Rica Powered by Arizona State University, San Jose 11501, Costa Rica; sierrahelen7@gmail.com; 6 Infectious Disease and Pulmonary Consultants, Victoria, TX 77901, USA; joanne.corderoguerra@gmail.com; 7 Texas A&M Rural Medicine, El Campo Memorial Hospital, Victoria, TX 77437, USA; msh.xatracho@gmail.com; 8 Instituto de Investigaciones One Health, Universidad Tecnológica Centroamericana, Tegucigalpa 11101, Honduras; hhesse.neurologia@gmail.com; 9 Department of Hospital Medicine, El Campo Memorial Hospital, El Campo, TX 77437, USA

**Keywords:** varicella-zoster virus, VZV, meningoencephalitis, autonomic dysfunction, neurogenic bladder, bowel dysmotility, neurotropism, immunocompetent host, sacral herpes zoster, neuroinvasive disease

## Abstract

Background: Varicella-zoster virus (VZV) is a neurotropic alphaherpesvirus capable of causing a broad spectrum of neurologic complications beyond classic dermatomal herpes zoster. Although meningitis and encephalitis are well recognized manifestations of neuroinvasive VZV infection, associated autonomic dysfunction remains comparatively underreported, particularly in immunocompetent individuals. Case Presentation: We describe a 66-year-old immunocompetent man who developed VZV meningoencephalitis associated with sacral dermatomal herpes zoster, urinary retention, and bowel dysmotility. Initial symptoms included fever, severe headache, photophobia, and low back pain, with delayed recognition of the characteristic sacral vesicular eruption. The patient subsequently developed encephalopathy and meningeal signs requiring intensive care unit admission. Cerebrospinal fluid analysis demonstrated lymphocytic pleocytosis and markedly elevated protein concentration, and VZV DNA was detected by polymerase chain reaction testing. During hospitalization, the patient developed severe urinary retention and gastrointestinal dysmotility without evidence of mechanical obstruction, raising concern for concurrent autonomic nervous system involvement. Following intravenous acyclovir therapy and supportive management, the patient experienced gradual neurologic and autonomic recovery. Conclusions: This case highlights the potential for multifocal neuroinvasive VZV disease involving both central and autonomic nervous system structures in immunocompetent hosts. Clinicians should maintain awareness that urinary retention and bowel dysmotility may represent clinically significant autonomic manifestations of VZV reactivation, particularly in the setting of sacral dermatomal involvement.

## 1. Introduction

Varicella-zoster virus (VZV), also known as human herpesvirus 3, is a neurotropic alphaherpesvirus capable of establishing lifelong latency following primary infection [1]. After resolution of primary varicella infection, the virus persists within dorsal root, cranial nerve, and autonomic ganglia, where it may remain dormant for decades before reactivation as herpes zoster [2]. Although herpes zoster classically presents as a painful unilateral dermatomal vesicular eruption, VZV reactivation may also result in a broad spectrum of neurologic complications including meningitis, meningoencephalitis, vasculopathy, cranial neuropathies, myelitis, necrotizing retinitis, and post-herpetic neuralgia [3,4,5,6,7,8,9]. While these complications occur more frequently in immunocompromised hosts, severe neuroinvasive disease may also develop in immunocompetent individuals [9,10].

In contrast to the well-recognized central nervous system manifestations of VZV, autonomic nervous system involvement remains comparatively underrecognized and likely underreported [11]. Reported autonomic manifestations include urinary retention, gastrointestinal dysmotility, constipation, pseudo-obstruction, and segmental visceral dysfunction [11,12,13,14,15]. These findings are believed to reflect viral involvement of autonomic ganglia or adjacent neural pathways following reactivation within sensory ganglia [9,11] (Supplementary Figure S1). Sacral dermatomal involvement appears particularly relevant in cases associated with bladder and bowel dysfunction because of the close neuroanatomic relationship between sacral sensory ganglia and parasympathetic outflow to pelvic organs [16,17,18,19] (Supplementary Figure S2).

The pathophysiology underlying multifocal neuroinvasive VZV disease remains incompletely understood. Proposed mechanisms include direct viral spread along neural pathways, inflammatory injury within adjacent neural structures, and immune-mediated dysregulation following viral reactivation [8,9,20,21]. Increasing understanding of VZV latency, immune evasion, and host antiviral signaling pathways has helped explain the heterogeneous clinical manifestations observed in some patients, including atypical neurologic and visceral involvement [22,23,24,25,26,27,28,29,30].

Here, we present a case of VZV meningoencephalitis occurring concurrently with urinary retention and bowel dysmotility in an immunocompetent patient with sacral dermatomal herpes zoster. This case highlights the extensive neurotropism of VZV and underscores the importance of recognizing autonomic manifestations as potential components of a unified neuroinvasive process.

## 2. Case Presentation

A 66-year-old previously healthy man presented to a local emergency department with a three-day history of fever, severe headache, photophobia, fatigue, and low back pain. In retrospect, the patient recalled the presence of a unilateral vesicular rash involving the right gluteal region at the time of his initial symptoms. Initial emergency department evaluation focused primarily on the headache. Computed tomography (CT) of the head was unremarkable, and the patient was discharged with a presumed diagnosis of migraine headache.

The following day, because of persistent low back pain and worsening constitutional symptoms, his primary care physician obtained lumbar magnetic resonance imaging (MRI) and additional CT imaging. Lumbar MRI demonstrated findings initially interpreted as possible L2–L3 spondylodiscitis. The patient was referred to a tertiary care emergency department for further evaluation; however, these findings were ultimately considered nonspecific and not fully explanatory of the patient’s evolving clinical presentation. Therefore, the patient was again discharged with a presumed viral syndrome and headache.

**Figure 1:**
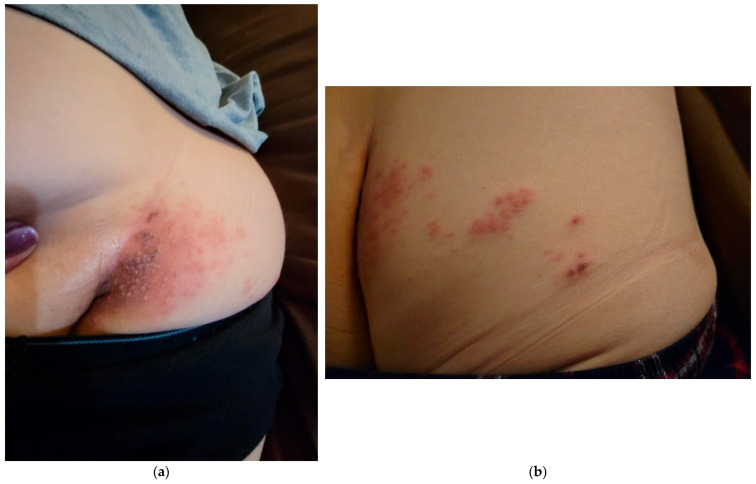
(**a**,**b**) Vesicular eruption involving the right gluteal region in a dermatomal distribution, consistent with herpes zoster. The distribution is most compatible with sacral dermatomes (likely S2–S3). The rash was first identified on day 6 of symptoms. Images provided by the patient.

On day 6 of illness, a unilateral dermatomal vesicular eruption extending from the gluteal cleft to the right buttock was noted (Figure 1a,b), and oral valacyclovir was initiated for presumed herpes zoster. The dermatomal distribution was most compatible with sacral involvement, likely involving the S2–S3 dermatomes. Despite antiviral therapy, the patient’s condition continued to deteriorate. By day 8 of illness, he developed encephalopathy, worsening headache, and meningeal symptoms, prompting transfer to our institution for higher-level care.

On admission to the intensive care unit, physical examination demonstrated photophobia, nuchal rigidity, bilateral hyperreflexia, positive Kernig sign, bilateral Babinski responses, and a vesicular sacral dermatomal rash concerning for neuroinvasive varicella-zoster virus infection. Intravenous acyclovir was initiated empirically following Infectious Disease consultation at a dose of 10 mg/kg every 8 h with pre- and post-saline hydration to avoid deposition crystallopathy. Cerebrospinal fluid (CSF) analysis demonstrated lymphocytic pleocytosis (300 cells/µL), markedly elevated protein concentration (716 mg/dL), and a CSF-to-serum glucose ratio of 0.53. CSF viral testing using Biofire Meningitis/Encephalitis Multiplex PCR panel (Biofire Diagnostics, Salt Lake City, UT, USA) detected VZV DNA, confirming the diagnosis of VZV meningoencephalitis [3,4,31].

Comprehensive evaluation was performed to assess alternative causes of meningoencephalitis and possible spinal cord involvement. Blood and CSF cultures remained negative. HIV testing was negative. The patient had no history of immunosuppressive medication exposure, biologic therapy, malignancy, chemotherapy, organ transplantation, chronic corticosteroid use, or known immunodeficiency [10]. Dedicated thoracic spinal imaging, formal autonomic testing, and urodynamic evaluation were not performed during hospitalization, limiting definitive characterization of potential radiculitis, myelitis, or autonomic pathway involvement.

**Figure 2:**
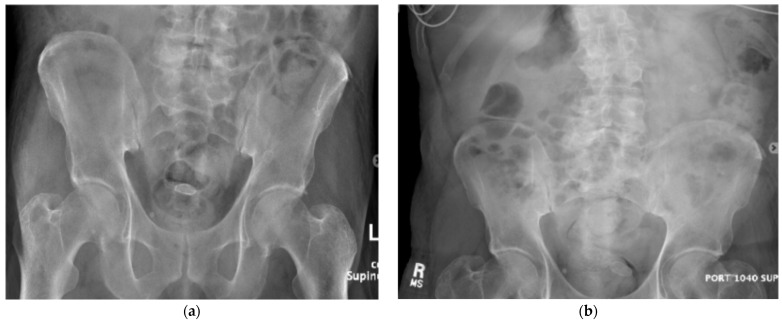
(**a**,**b**) Serial abdominal radiographs during hospitalization. (**a**) Day 8. Supine abdominal radiograph demonstrating moderate fecal retention with gaseous distention of the colon, most prominent in the ascending colon and rectosigmoid region. No radiographic evidence of mechanical bowel obstruction is identified. (**b**) Day 16. Follow-up abdominal radiograph demonstrating persistent fecal retention and gaseous distention without evidence of mechanical bowel obstruction. An incidental radiopaque density in the left renal region is suggestive of nephrolithiasis. Lumbar levoscoliosis and degenerative changes in the lumbar spine are also noted.

During hospitalization, progressive abdominal distention and urinary retention prompted further evaluation. Bladder scan demonstrated approximately 1100 mL of retained urine requiring Foley catheter placement. Abdominal radiography demonstrated diffuse gaseous bowel distention and fecal retention without evidence of mechanical obstruction (Figure 2a,b). The coexistence of urinary retention and bowel dysmotility raised concern for concurrent autonomic nervous system involvement affecting pelvic visceral function in the setting of sacral dermatomal VZV reactivation [11,12,13,14,15,16,17,18,19].

Following stabilization in the intensive care unit, the patient was transferred to a long-term acute care facility for ongoing management and rehabilitation. Persistent urinary retention failed to improve with tamsulosin therapy, requiring continued catheter-based bladder management and bladder training. Severe constipation and bowel dysmotility were initially refractory to standard laxative therapy. He was subsequently given a 3-day course of erythromycin at a dose of 500 mg three times daily for its prokinetic properties, resulting in improvement in bowel function within approximately two days [13]. He completed a 21-day course of intravenous acyclovir based on a combination of published recommendations for neuroinvasive VZV infection, specialist judgement as the patient had seven days of symptoms with no treatment, and clinical response [31].

**Figure 3:**
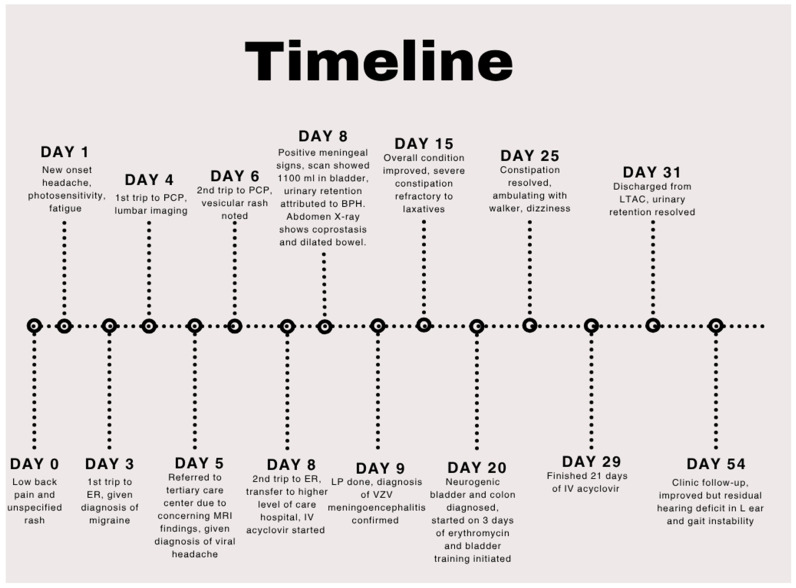
Clinical timeline illustrating the progression of symptoms, diagnostic evaluation, and treatment, with key events shown relative to symptom onset. Abbreviations: ER, emergency department; PCP, primary care physician; MRI, magnetic resonance imaging; IV, intravenous; BPH, benign prostatic hyperplasia; LP, lumbar puncture; VZV, varicella-zoster virus; LTAC, long-term acute care facility; L, left.

Over the subsequent weeks, the patient demonstrated gradual neurologic and autonomic recovery. Urinary retention progressively improved and ultimately resolved prior to follow-up. At one-month outpatient follow-up, the patient demonstrated improved gait stability, near-complete recovery of bowel and bladder function, and mild residual left-sided hearing impairment with subtle persistent alterations in interoceptive awareness. A clinical timeline summarizing the progression of symptoms, diagnostic evaluation, and treatment course is presented in Figure 3.

## 3. Discussion

### 3.1. Neuroinvasive Varicella-Zoster Virus Infection in Immunocompetent Hosts

Varicella-zoster virus (VZV) is a highly neurotropic alphaherpesvirus capable of establishing lifelong latency within sensory, cranial nerve, and autonomic ganglia following primary infection [1,2,9]. Reactivation classically manifests as herpes zoster; however, the neurologic spectrum of VZV infection extends far beyond dermatomal skin involvement and includes meningitis, meningoencephalitis, vasculopathy, cranial neuropathies, myelitis, necrotizing retinitis, and stroke syndromes [3,4,5,6,7,8]. Although severe neuroinvasive disease occurs more commonly in immunocompromised patients, increasingly recognized evidence demonstrates that substantial neurologic morbidity may also develop in immunocompetent individuals [8,9,11].

In the present case, the patient developed confirmed VZV meningoencephalitis in association with sacral dermatomal herpes zoster despite the absence of known immunodeficiency, chronic corticosteroid exposure, malignancy, transplantation, biologic therapy, or HIV infection. This presentation highlights the importance of maintaining clinical suspicion for neuroinvasive VZV even in patients without traditional immunosuppressive risk factors. Importantly, the patient’s rash was initially overlooked and early symptoms were interpreted as migraine and nonspecific viral illness, contributing to delayed recognition of the evolving neuroinfectious process.

### 3.2. Autonomic Manifestations and Sacral Neuroanatomic Localization

Compared with the well-characterized central nervous system manifestations of VZV, autonomic complications remain comparatively underrecognized and likely underreported [11]. Reported autonomic manifestations include urinary retention, constipation, gastrointestinal dysmotility, pseudo-obstruction, erectile dysfunction, and segmental visceral dysfunction [11,12,13,14,15]. These complications are believed to reflect viral involvement of autonomic ganglia or adjacent autonomic pathways following reactivation within sensory ganglia.

The neuroanatomic distribution observed in this patient is particularly notable. The unilateral sacral dermatomal eruption localized predominantly to the S2–S3 distribution, anatomically adjacent to parasympathetic outflow supplying the bladder, distal colon, and rectum [16,17,18,19] (Supplementary Figure S2; Supplementary Discussion S2). During hospitalization, the patient developed severe urinary retention with approximately 1100 mL of retained urine in addition to bowel dysmotility without radiographic evidence of mechanical obstruction. The temporal relationship between sacral dermatomal reactivation and concurrent pelvic visceral dysfunction raises concern for simultaneous autonomic nervous system involvement affecting pelvic organ function.

Prior reports have described urinary retention associated with sacral herpes zoster, including cases accompanied by meningitis and erectile dysfunction [14]. Similarly, gastrointestinal dysmotility and pseudo-obstruction related to VZV infection have been reported in both immunocompetent and immunocompromised patients [12,13,15]. To our knowledge, no prior cases describing simultaneous VZV meningoencephalitis with concurrent urinary retention and bowel dysmotility in an immunocompetent patient were identified during our review of the English-language literature. Although individual manifestations have been separately recognized, the coexistence of central nervous system infection and clinically significant autonomic dysfunction suggests a broader multifocal neuroinvasive phenotype than is commonly appreciated.

### 3.3. Concurrent Central and Autonomic Nervous System Involvement

The coexistence of meningoencephalitis, urinary retention, and bowel dysmotility in this patient raises the possibility of concurrent involvement of both central and autonomic nervous system structures during VZV reactivation. One possible explanation is that viral reactivation within sacral sensory ganglia extended into adjacent autonomic pathways supplying pelvic viscera (Supplementary Figures S1 and S2). Alternative contributing mechanisms may include localized inflammatory injury, viral radiculitis, or immune-mediated neural dysfunction associated with neuroinvasive VZV infection [8,9,20,21]. The differential diagnosis during hospitalization included acute transverse myelitis, sacral radiculitis (including Elsberg syndrome), spinal cord infarction, and other inflammatory demyelinating disorders such as neuromyelitis optica spectrum disorder and MOG antibody-associated disease. Acute transverse myelitis could not be definitively excluded because dedicated thoracic spinal imaging and formal neurophysiologic evaluation were not performed. However, the temporal association with sacral dermatomal herpes zoster, positive CSF VZV PCR testing, and gradual improvement following antiviral therapy supported a unifying neuroinvasive VZV-associated process.

Importantly, definitive characterization of the underlying neurologic process was limited by the absence of dedicated thoracic spinal imaging, formal autonomic testing, electromyography, and urodynamic evaluation. Consequently, the presence of transverse myelitis, radiculitis, or specific autonomic pathway involvement cannot be conclusively established. Nevertheless, the temporal association between sacral dermatomal herpes zoster, confirmed VZV meningoencephalitis, severe urinary retention, and bowel dysmotility strongly supports a unified neuroinvasive process rather than multiple unrelated clinical entities.

This case also highlights the distinction between visceral autonomic dysfunction and disseminated visceral organ involvement in VZV infection. Disseminated visceral VZV disease may involve organs such as the lungs, liver, or myocardium and has been associated with substantial morbidity and mortality, particularly among immunocompromised patients [32]. In contrast, the present patient demonstrated functional visceral dysregulation manifested by neurogenic bladder and bowel dysfunction without evidence of direct visceral organ infection or disseminated cutaneous disease. Recognition of this distinction is clinically important because visceral symptoms in patients with herpes zoster may reflect either direct organ involvement or neurogenic autonomic dysfunction.

### 3.4. Diagnostic Lessons and Clinical Implications

This case illustrates the potential consequences of fragmented clinical reasoning during the early evaluation of neuroinfectious disease. The patient initially presented with fever, severe headache, photophobia, and low back pain, yet these findings were evaluated primarily as isolated complaints rather than as components of a unified neurologic process. New-onset migraine-like headache in an older adult is comparatively uncommon and should prompt careful consideration of secondary etiologies, particularly when accompanied by systemic symptoms or neurologic findings [33].

Recognition of the sacral dermatomal eruption ultimately provided an important diagnostic clue; however, the subsequent development of encephalopathy and meningeal signs was required before neuroinvasive VZV infection was strongly considered. The autonomic manifestations proved similarly important. Severe urinary retention and bowel dysmotility occurring simultaneously with sacral dermatomal herpes zoster and central nervous system infection provided a coherent neuroanatomic framework linking otherwise seemingly disparate clinical findings.

This case further emphasizes that neuroinvasive VZV infection may evolve over several days and may initially present in incomplete or atypical forms. Clinicians should maintain awareness that autonomic dysfunction, including urinary retention and gastrointestinal dysmotility, may represent clinically significant manifestations of VZV neuroinvasion, particularly in the setting of sacral dermatomal involvement.

### 3.5. Mechanistic Considerations

The mechanisms underlying multifocal neuroinvasive VZV disease remain incompletely understood. Following primary infection, VZV establishes latency within sensory and autonomic ganglia with the potential for subsequent reactivation [2,22,23] (Supplementary Figures S1 and S3; Supplementary Discussions S1 and S3).

Current evidence suggests that neurologic injury in VZV infection likely reflects a combination of direct viral effects, inflammatory responses, and immune-mediated neural dysfunction [8,20,21]. Viral latency, reactivation, and host immune responses may contribute to the heterogeneous neurologic manifestations observed in some patients, including atypical presentations occurring in immunocompetent individuals [22,23,24,25,26,27,28,29,30].

Although the precise mechanism underlying the autonomic manifestations observed in this patient cannot be definitively established, the clinical course supports a temporally related multifocal neuroinvasive process associated with VZV reactivation. Further investigation into the relationship between sensory ganglionic reactivation, autonomic dysfunction, and central nervous system involvement may improve understanding of atypical VZV presentations and facilitate earlier recognition of these potentially underdiagnosed complications.

## 4. Conclusions

This case highlights concurrent central nervous system and autonomic nervous system involvement associated with varicella-zoster virus (VZV) reactivation in an immunocompetent patient. The temporal association between sacral dermatomal herpes zoster, confirmed VZV meningoencephalitis, urinary retention, and bowel dysmotility supports a multifocal neuroinvasive process involving both sensory and autonomic pathways. Although definitive autonomic testing and dedicated spinal imaging were not performed, the clinical course and response to antiviral therapy strongly suggest clinically significant autonomic dysfunction associated with neuroinvasive VZV infection.

This report additionally underscores that autonomic manifestations of VZV, including urinary retention and gastrointestinal dysmotility, may be underrecognized in clinical practice, particularly when presenting alongside more prominent neurologic symptoms. Recognition of these findings may facilitate earlier diagnostic unification and more timely initiation of antiviral therapy. Importantly, visceral symptoms in patients with herpes zoster may reflect neurogenic autonomic dysfunction rather than disseminated visceral organ infection alone.

Overall, this case emphasizes the extensive neurotropism of VZV and the importance of maintaining clinical suspicion for atypical neuroinvasive presentations, even in patients without recognized immunocompromising conditions.

## Data Availability

The original contributions presented in this study are included in the article/Supplementary Material. Further inquiries can be directed to the corresponding author.

## Supplementary Materials

The following supporting information can be downloaded at https://www.mdpi.com/article/10.3390/idr18030058/s1: Supplementary Table S1. Human Herpesviruses and Selected Neurologic and Systemic Manifestations. Supplementary Figure S1. Pathogenesis and Behavior of Varicella-Zoster Virus. Supplementary Discussion S1. Neurotropism, Latency, and Reactivation. Supplementary Figure S2. Simplified Schematic of Autonomic Innervation of the Distal Gastrointestinal Tract and Bladder. Supplementary Discussion S2. Neuroanatomic Considerations in Autonomic Dysfunction. Supplementary Figure S3. Varicella-Zoster Virus Replication Cycle and Transcriptional Program. Supplementary Discussion S3. Molecular Mechanisms of Latency, Reactivation, and Immune Evasion. References [34,35,36,37,38,39,40,41,42] are cited in the supplementary Materials.

## Abbreviations

The following abbreviations are used in this manuscript:

CNS	Central nervous system
DNA	Deoxyribonucleic acid
GI	Gastrointestinal
ORF	Open reading frame
PNS	Peripheral nervous system
RNA	Ribonucleic acid
S2–S4	Sacral nerve roots 2 through 4
VLT	VZV latency-associated transcript
VZV	Varicella-zoster virus
