# Supplementary material for: Concurrent Central and Autonomic Nervous System Involvement in Varicella-Zoster Virus Infection in an Immunocompetent Patient: A Case-Based Mechanistic Analysis
Source: Infect Dis Rep. 2026 Jun 15;18(3):58. doi: 10.3390/idr18030058 (PMC13299381; doi:10.3390/idr18030058)
Supplement: Supplementary file 1 [file idr-18-00058-s001.zip › idr-4274006-supplementary.pdf]

Concurrent Central and Autonomic Nervous System Involvement in Varicella-Zoster Virus Infection in an Immunocompetent Patient: A Case-Based Mechanistic Analysis

Supplementary Materials

Supplementary Table S1. Human Herpesviruses and Selected Neurologic and Systemic Manifestations

Alpha Subfamily		Classic Presentation
HHV-1	Herpes Simplex Virus 1	Recurrent lip ulcers, meningitis, meningoencephalitis
HHV-2	Herpes Simplex Virus 2	Recurrent painful genital ulcers, meningitis, meningoencephalitis
HHV-3	Varicella-Zoster Virus	Children - Primary varicella (chickenpox)<br>Adults - Herpes zoster (shingles)<br>Pneumonia, Myocarditis
**Beta Subfamily**		
HHV-5	Cytomegalovirus	Immunocompetent patients - Syndrome similar to mononucleosis<br>Immunosuppressed patients - Multiorgan dysfunction
HHV-6	Human Herpesvirus 6	Children - Roseola<br>Adults - Fever and rash
HHV-7	Human Herpesvirus 7	Benign, self-limited, no known robust clinical syndrome
**Gamma Subfamily**		
HHV-4	Epstein Barr Virus	Mononucleosis, Lymphoma
HHV-8	Kaposi's Sarcoma Associated Herpesvirus	Kaposi's sarcoma, Castleman disease, Primary Effusion Lymphoma. Kaposi Sarcoma Inflammatory Cytokine Syndrome

Legend:
Representative clinical manifestations associated with major human herpesviruses. The table is intended as a concise contextual overview relevant to the neurotropic behavior of varicella-zoster virus discussed in the present report.

Supplementary Figure S1. Pathogenesis and Neuroinvasive Behavior of Varicella-Zoster Virus

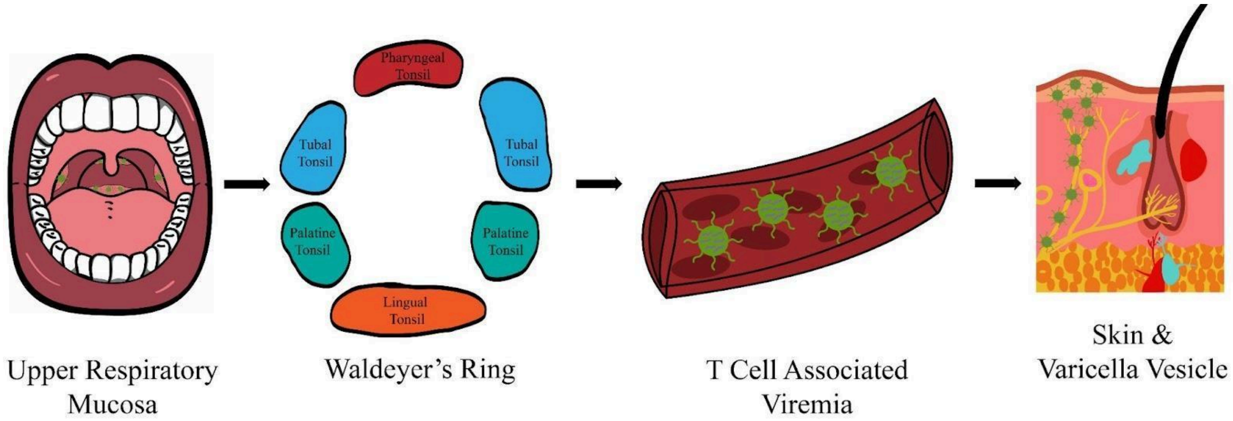

A

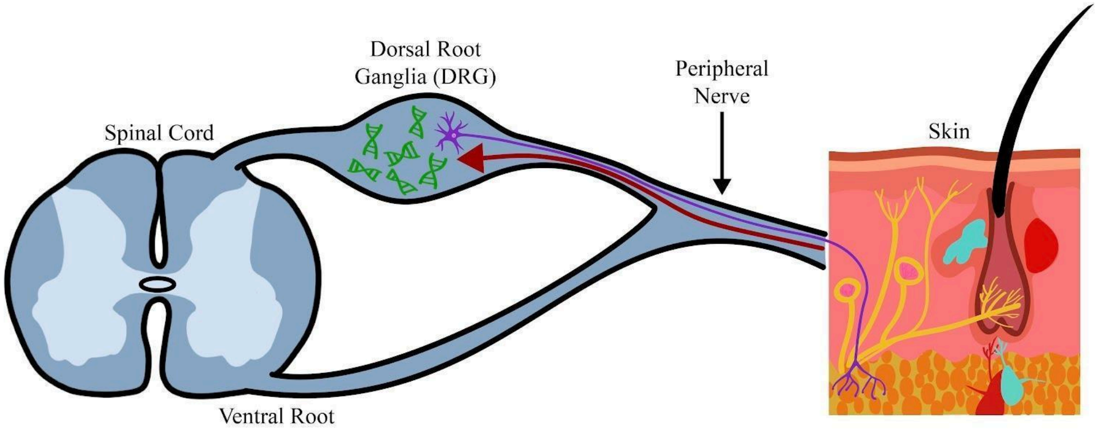

B

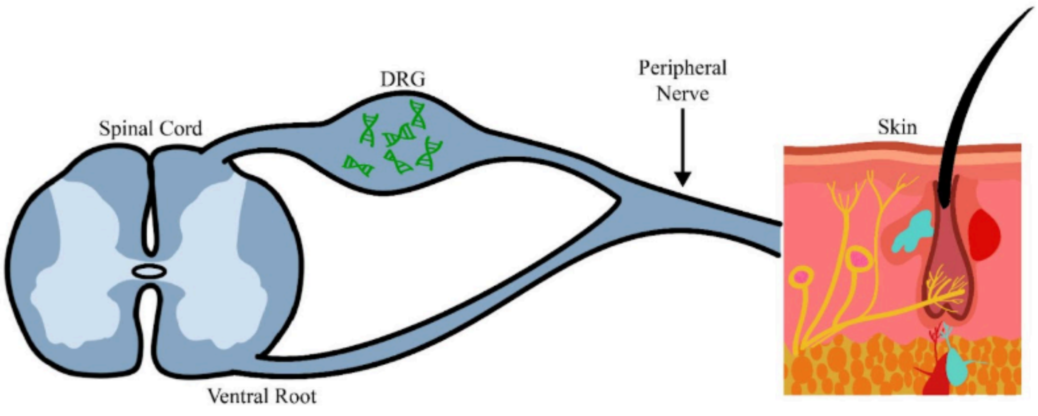

C

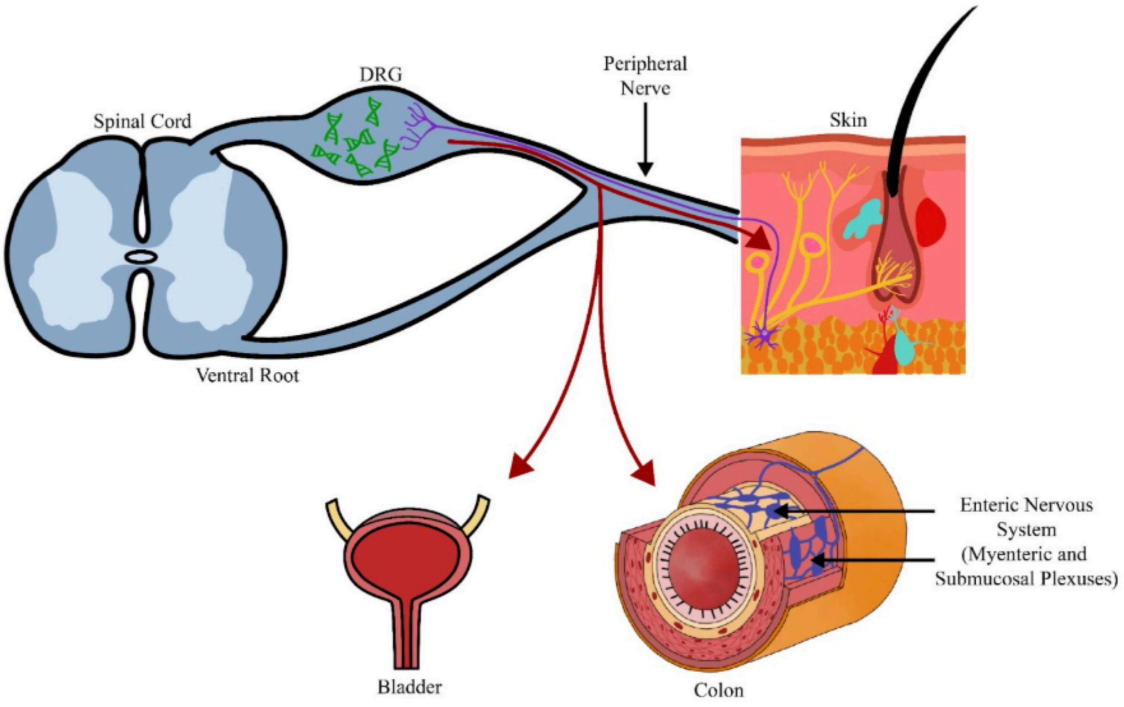

D

Legend:

(A) Primary VZV infection begins following inoculation of the upper respiratory mucosa with subsequent replication within lymphoid tissue, including Waldeyer's ring. T-cell–associated viremia facilitates dissemination to the skin, resulting in varicella lesions. (B) Following primary infection, VZV undergoes retrograde axonal transport to dorsal root ganglia (DRG), where latent infection is established. (C) During latency, VZV persists within sensory ganglia in a transcriptionally restricted state characterized by minimal viral gene expression and absence of productive viral replication. (D) Reactivation of latent VZV

results in anterograde axonal transport along peripheral nerves with spread to the skin producing dermatomal herpes zoster. In some cases, viral spread or associated inflammatory processes may extend beyond sensory pathways to involve the central nervous system and autonomic nervous system structures, including pathways supplying the bladder and gastrointestinal tract.

Supplementary Discussion S1. Neurotropism, Latency, and Reactivation

Varicella-zoster virus demonstrates marked neurotropism with the capacity to establish lifelong latency following primary infection [2,9,20]. During primary infection, viral replication within respiratory mucosa and lymphoid tissue is followed by dissemination through infected T lymphocytes, permitting spread to the skin and nervous system [1,20,34]. After resolution of primary infection, VZV persists within dorsal root ganglia, cranial nerve ganglia, and autonomic ganglia where it may remain dormant for decades before reactivation.

Latency is increasingly understood as a transcriptionally restricted but biologically active state rather than complete viral quiescence [22,23,35–37]. Reactivation may occur in association with aging, impaired immune surveillance, neuronal stress, or other incompletely characterized host factors [9,10]. Following reactivation, viral spread along sensory pathways results in the characteristic dermatomal manifestations of herpes zoster. In selected patients, however, viral spread and associated inflammatory responses may involve central nervous system and autonomic nervous system structures, producing atypical neurologic and visceral manifestations [3,8,11].

Supplementary Figure S2. Autonomic Innervation of the Distal Gastrointestinal Tract and Bladder

Autonomic Innervation of the Distal Gastrointestinal Tract and Bladder

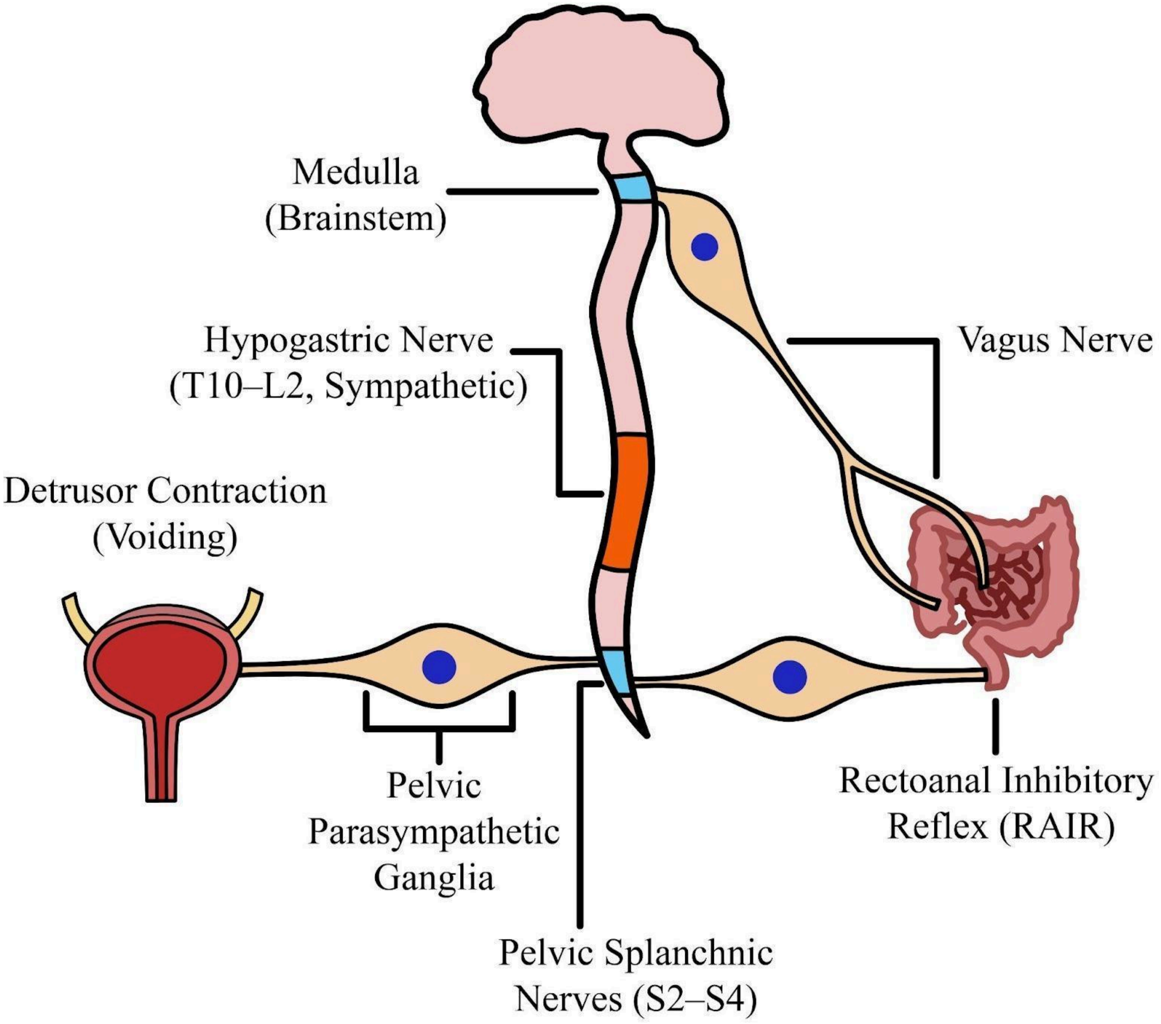

Legend:
Simplified schematic illustrating autonomic innervation of the distal gastrointestinal tract and bladder. Parasympathetic innervation to distal pelvic organs arises predominantly from sacral segments S2–S4 through the pelvic splanchnic nerves and contributes to detrusor contraction, rectal motility, and defecatory reflexes including the rectoanal inhibitory reflex (RAIR). Sympathetic innervation from thoracolumbar segments T10–L2 via the hypogastric nerve contributes primarily to urinary and fecal storage functions. Vagal parasympathetic innervation supplies the proximal colon to approximately the splenic flexure.

Supplementary Discussion S2. Neuroanatomic Considerations in Autonomic Dysfunction

The autonomic nervous system regulates involuntary visceral function through coordinated sympathetic and parasympathetic pathways [16–19,38]. Sacral parasympathetic outflow from spinal segments S2–S4 mediates bladder emptying and distal gastrointestinal motility, whereas sympathetic pathways originating from thoracolumbar segments promote urinary and fecal storage [16–19].

In cases of sacral dermatomal herpes zoster, close anatomic proximity between sensory ganglia and pelvic autonomic pathways may permit extension of viral inflammation or associated neural dysfunction involving bladder and gastrointestinal innervation [11,16–19]. This proposed neuroanatomic relationship may help explain autonomic manifestations including urinary retention, constipation, bowel dysmotility, and pseudo-obstruction reported in association with sacral VZV reactivation [11–15].

Supplementary Figure S3. Varicella-Zoster Virus Replication Cycle and Transcriptional Program

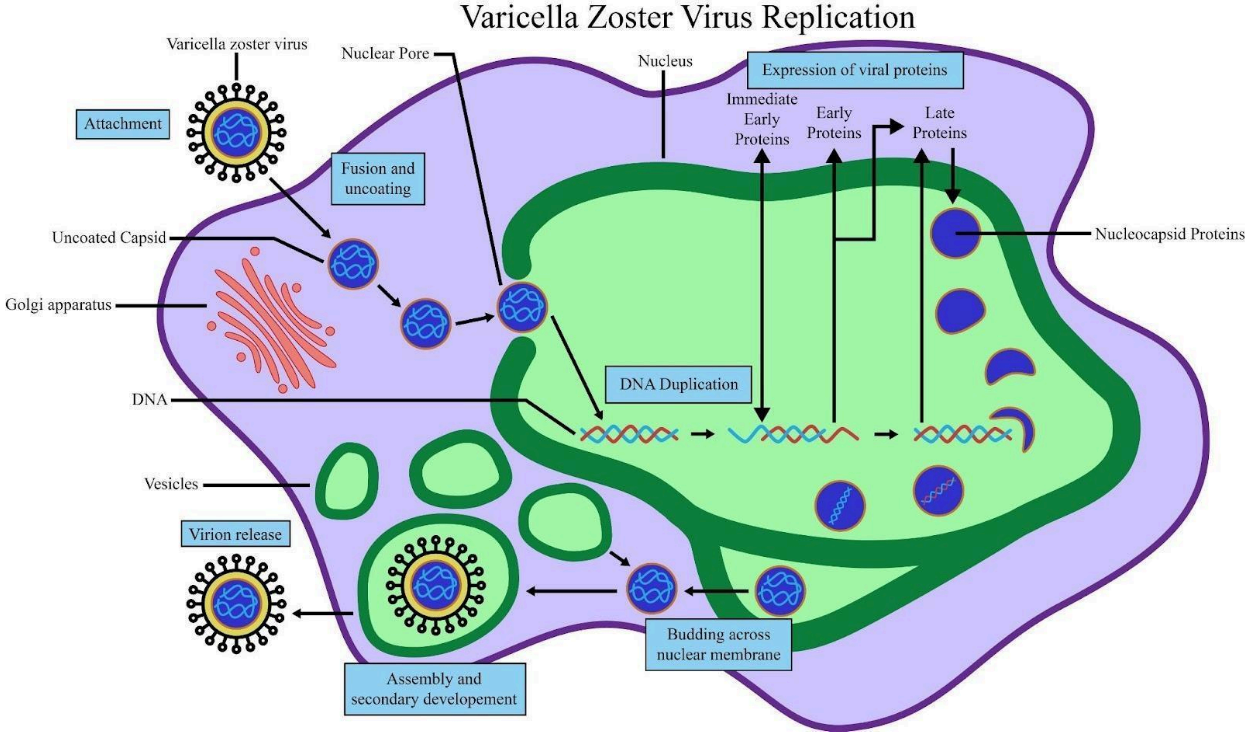

Legend:
Schematic representation of the varicella-zoster virus replication cycle. Infection begins with viral attachment to the host-cell membrane followed by fusion and uncoating. The viral capsid is transported to the nucleus where viral DNA is released and undergoes replication. Viral gene expression proceeds through coordinated immediate-early, early, and late transcriptional phases, resulting in synthesis of regulatory and structural proteins. Newly formed nucleocapsids assemble within the nucleus, acquire envelope components through budding across the nuclear membrane and Golgi-associated processing, and are subsequently released to infect neighboring cells.

Supplementary Discussion S3. Molecular Mechanisms of Latency, Reactivation, and Immune Evasion

The VZV genome consists of a linear double-stranded DNA molecule encoding more than 70 open reading frames [22,39]. During latency, viral DNA persists predominantly in episomal configuration within neuronal tissue and demonstrates highly restricted transcriptional activity [22,40,41]. Increasing evidence suggests that latency is regulated through complex interactions between viral transcriptional programs, epigenetic regulation, chromatin structure, and host immune surveillance [21–23,35–37,42].

Reactivation is associated with renewed viral gene expression and progression through immediate-early, early, and late transcriptional phases required for productive replication [35–37]. Host antiviral control relies heavily on interferon-mediated signaling pathways and T-cell–mediated immune surveillance [24–30]. VZV possesses multiple immune evasion mechanisms, including disruption of interferon signaling and modulation of host antiviral responses [24,27–30]. These interactions may contribute to the heterogeneous neurologic manifestations observed during VZV reactivation, including atypical multifocal neuroinvasive presentations involving both central and autonomic nervous system structures.
